# Acute effects of an isometric neck warm-up programme on neck performance characteristics and ultrasound-based morphology

**DOI:** 10.1080/07853890.2023.2295402

**Published:** 2023-12-23

**Authors:** Takashi Nagai, Nathan D. Schilaty, Hanwen Wong, Valerie C. Keller, Sean T. Stiennon, Ryan W.B Chang, Michael J. Stuart, David A. Krause

**Affiliations:** a United States Army Research Institute and Environmental Medicine, Natick, MA, USA; b Department of Orthopedic Surgery, Mayo Clinic, Rochester, MN, USA; c Department of Neurosurgery and Brain Repair, University of South FL, Tampa, FL, USA; d Department of Medical Engineering, University of South Florida, Tampa, FL, USA; e Center for Neuromusculoskeletal Research, University of South Florida, Tampa, FL, USA; f Department of Physical Medicine & Rehabilitation, Mayo Clinic, Rochester, MN, USA

**Keywords:** Isometric, warm-up, cervical, neck, force development, proprioception, sensorimotor, performance enhancement

## Abstract

**Objective:**

Athletic performance can be enhanced immediately after an isometric warm-up, a phenomenon termed post-activation performance enhancement (PAPE). While isometric warm-ups can improve lower extremity sprint and jump performance, neck-specific isometric warm-ups need development and validation for mild traumatic brain disorders and neck pain. This study examined acute effects of isometric warm-ups on neck performance and morphology.

**Methods:**

*Arm 1:* Twenty-six adults (13 M:13F) completed neck performance testing before and after a 10-minute neck isometric warm-up or stationary bike (sham) between two visits. Testing included visual-motor reaction time, peak force, rate of force development, force steadiness, and force replication/proprioception measured by a 6-axis load cell. An inclinometer assessed range-of-motion. Paired t-tests and two-way ANOVA examined effects of neck/bike warm-up and interaction effects, respectively. *Arm 2:* 24 adults (11 M:13F) completed ultrasound scans of cervical muscles: before 20-minute rest (sham), and before/after a 5-min neck isometric warm-up. Longus colli cross-sectional area and sternocleidomastoid/upper trapezius thickness and stiffness, and cervical extensors thickness was assessed. One-way ANOVA compared morphological values at sham, before, and after warm-up. Significance was set at *p* < 0.05.

**Results:**

Isometric neck warm-up increased rate of force development in flexion (*p* = 0.022), extension (*p* = 0.001–0.003), right lateral flexion (*p* = 0.004–0.032), left lateral flexion (*p* = 0.005–0.014), while peak force improved only in left lateral flexion (*p* = 0.032). Lateral flexion range-of-motion increased after neck warm-up (*p* = 0.003-0.026). Similarly, longus colli cross-sectional area (*p* = 0.016) and sternocleidomastoid thickness (*p* = 0.004) increased.

**Conclusions:**

Increased neck performance characteristics and morphology are likely due to PAPE effects of isometric neck warm-up. For coaches and athletes, simple isometric contractions could be added to existing warm-ups to reduce prevalence, incidence, and severity of mild traumatic brain injuries and neck pain.

## Introduction

There is an estimated incidence rate of 1.6–3.8 million mild traumatic brain injuries (mTBI) annually in the United States, potentially resulting in short and long-term physical, cognitive, behavioural, and emotional issues [1]. Total medical cost of mTBI was $40.6 billion in 2016 [2]. Individuals with mTBI can exhibit cervicogenic post-concussion syndrome including neck pain (NP), balance disturbance, headache, decreased neck range-of-motion, and impaired proprioception [3,4]. Those individuals with post-mTBI and NP are at a higher risk (2.6-6.4 times) of developing persistent post-concussive symptoms than individuals with post-mTBI without NP, and face longer recovery time [3]. Therefore, primary prevention strategies such as better protective gear, policy/rule changes, and training strategies (i.e. neck neuromuscular training) have been widely discussed [5].

Specifically, a simple neck strengthening exercise with neck isometric contractions (10-second hold in flexion, extension, and lateral flexions) was added to a warm-up routine; and youth rugby players who did the exercise programme had fewer head and neck injuries [6]. Similarly, a simple 30-second isometric neck-hold in flexed (chin tucked) and rotated (right and left rotation) positions while rolling backward and forward on their back (∼90 s total per session for 2–4 times a week for an entire season) were added to a soccer warm-up routine; and youth soccer players who did this simple exercise programme had fewer mTBI incidence [7]. These isometric contractions are shown to temporarily optimize (prepare) individual’s neuromuscular characteristics and athletic performance: this phenomenon is known as ‘post-activation potentiation’ or ‘post-activation performance enhancement (PAPE)’ [8].

Since the majority of PAPE studies focused on individuals’ athletic performance enhancement such as jumps, sprints, and throws [9], it is largely unknown if simple isometric contractions of neck musculatures would enhance individual’s neck neuromuscular characteristics such as peak force and rate of force development. Since these neck neuromuscular characteristics are shown to reduce brain injury risk (based on simulation models) [10,11], it is clinically significant to develop a neck warm-up programme that is capable of inducing neck-specific PAPE.

In addition, there are several neck-specific sensorimotor characteristics and morphology, that are often included as a part of clinical tests in patients with mTBI and NP. For example, individual’s ability to visually perceive a head impact and react quickly (i.e. visual-motor reaction time) is associated with lower linear and angular head acceleration, potentially mitigating the incidence and severity of concussion [11]. Diminished neck range-of-motion and proprioception were commonly observed in individuals with NP [12]. Individuals with NP also exhibited diminished ability to maintain neck force steadiness, resulting a larger variability (increased coefficient of variation during force steadiness task) [13]. Anatomically, deep neck muscles including the longus colli and cervical multifidi are thought to play a critical role in neck stability in individuals with NP [14]. The cross-sectional area and stiffness of these neck muscles can be measured using diagnostic ultrasound with shear wave elastography [15].

Therefore, the primary purpose of the study was to investigate immediate main effects of a neck-specific warm-up programme on neck performance charac­teristics (neck visual-motor reaction time, peak force, rate of force development, force steadiness, force replication sense, and range-of-motion) as well as interaction effects of the neck warm-up programme vs. bike (sham) warm-up in a cross-over research design with two separate laboratory visits. The secondary purpose of the study was to examine the immediate main effects of neck flexion and extension isometric contractions on morphology of the cervical muscles (cross-sectional area, thickness, and stiffness) when compared to the rest (sham) intervention within one laboratory visit. It was hypothesized that a neck warm-up programme would induce favourable changes in neck performance (increased peak force, rate of force development, range-of-motion, faster reaction time, and better proprioception) and morphology of cervical musculature (increased muscle cross-sectional area, thickness, and stiffness).

## Methods

### Experimental approach – effects of the COVID-19 pandemic

**Figure 1.**
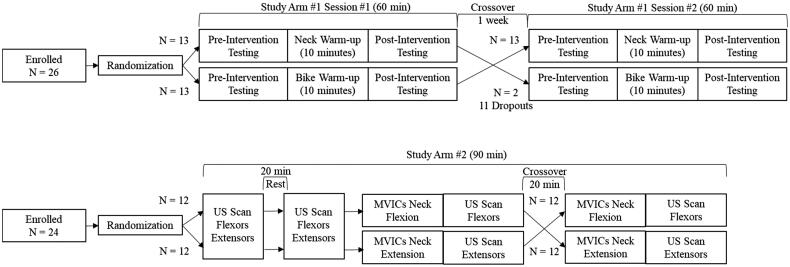
A Crossover research design. Top and bottom figure is study arm #1 and arm #2, respectively.

This project was continuation of a previous study that established reliability of ultrasound-based measurements of neck muscle thickness and neck sensorimotor motor characteristics [15–17]. As stated earlier, this manuscript has two purposes. Two separate study arms were used to examine the main effects of a neck-warm-up programme with isometric contractions on neck performance (study arm #1) and neck morphology (study arm #2). For study arm #1, a repeated-measures cross-over research design with 2 separate laboratory visits (∼1 week apart) were used to measure neck performance characteristics before and after 2 different warm-up programmes (a. neck warm-up vs. b. bike [sham] warm-up). The order of neck warm-up and bike warm-up were randomized for the first 15 subjects. However, prior to the COVID-19 laboratory shutdown, 11 subjects were asked to complete the neck warm-up first, given unforeseen circumstance (in fact, these 11 subjects were not allowed to return to the campus during the COVID-19 pandemic, therefore, the number of participants who completed the bike warm-up (*n* = 15) were fewer than the number of participants who completed the neck warm-up (*n* = 26). For study arm #2, the same repeated-measures cross-over research design was used; however, 2 separate interventions (a. neck warm-up vs. b. control/rest [sham]) were evaluated within one laboratory visits. Ultrasound scans were performed three times within 90 min (before and after 20 min rest and after a 5-min neck warm-up with isometric contractions). The neck warm-up with isometric contractions in the flexion direction were immediately followed by ultrasound scanning of the cervical flexors. Similarly, the neck warm-up with isometric contractions in the extension direction was immediately followed by ultrasound scanning of the cervical extensors. The order of flexion and extension contractions were randomized. The overall interventions, orders, and participants numbers for the study arm #1 and #2 are summarized in Figure 1.

Due to a global pandemic of coronavirus disease 2019 (COVID-19), our laboratory was partially shutdown during study arm #1. There were 26 participants who completed the neck warm-up while only 15 participants were able to do both neck and bike interventions. Therefore, the first primary purpose (effects of the neck warm-up programme on neck performance) was examined using a test-retest research design with 26 participants while the interaction effects of differences between two interventions (neck warm-up vs. sham warm-up) were examine using a 2 (time) x 2 (interventions) within-subjects analysis of variance design with 15 participants. For study arm #2, there were 24 participants who completed three testing sessions (1. pre-sham, 2. post-sham/pre-neck, 3. post-neck isometric contractions) within one laboratory visit. A one-way (time) within-subjects analysis of variance design was used. This study arm #2 was not affected by the COVID-19 laboratory shutdown.

### Subjects

The Institutional Human Ethics Review Board reviewed and approved the study. Informed consent was obtained from all participants. Inclusionary criteria included physically active individuals between the ages of 18 to 30 participating in exercise at least 3 times a week with no history of mTBI or NP. The international physical activity questionnaire short form was completed to assure all participants were in the moderate or high activity categories [18]. Participants completed the neck disability index and were excluded if any disability greater than 4 points was identified [19]. A screening evaluation consisted of active flexion, extension, side bending, rotation range-of-motion assessment and a Spurling’s compression test. Participants were excluded if they had current neck pain, past cervical pathology or surgery, or pain with the screening tests. They were also excluded if they had any condition limiting the ability to perform multi-directional neck isometric contractions. Based on an *a priori* power analysis, 21 or more participants provided over 80% power to detect a 0.5 SD (effect size Cohen’s *d* = 0.50) difference between pre- and post-intervention. Twenty-six (13 males and 13 females) and 24 (11 males and 13 females) participated in the study arm #1 and #2, respectively. As stated earlier, several individuals were unable to return to the laboratory testing due to the COVID-19 lockdown. Therefore, two demographics information (age, height, and weight) for study arm #1 (Visit 1 and Visit 2) as well as demographics information for study arm #2 are shown in Table 1.

**Table 1.** Demographics.

	Study Arm #1 Visit 1 (*n* = 26: 13f/13m)	Study Arm #1 Visit 2 (*n* = 15: 10f/5m)	Study Arm #2 (*n* = 24: 13f/11m)
Age, years	24.2 ± 1.7	24.5 ± 2.1	24.6 ± 3.6
Height, cm	171.8 ± 8.2	168.1 ± 7.4	172.4 ± 9.2
Weight, kg	72.3 ± 11.2	70.6 ± 11.3	70.7 ± 11.8

### Procedures study arm #1: Neck performance

For study arm #1, a cervical range-of-motion device (Hospeq, Miami, FL, USA) was used to measure active range-of-motion in the flexion, extension, right/left lateral flexions, and rotations. Participants were seated in a chair and the cervical range-of-motion device was positioned. The participant was asked to move their head as far as they could in the directions of flexion, extension, right/left lateral flexions, and rotations for three times as practice. Motions were repeated three times to record their range-of-motion. An average of three measurements was used for analysis. The intraclass correlation coefficients (ICCs) for intra-examiner reliability have been shown to be 0.39 to 0.95 [20]. Additionally, the cervical range-of-motion device has excellent criterion validity for cervical rotation and concurrent validity for flexion, extension, and lateral flexion measurements [21, 22].

A custom-built multidirectional neck isometric dynamometer testing device was used to collect neck visual-motor reaction time, peak force, and rate of force development, force steadiness, and force replication sense [16]. Participants were seated in the testing device, an adjustable harness to prevent trunk movement was fastened, and a helmet (Rawlings Coolflo Batting Helmet, Rawlings, St. Louis, MO, USA) was fitted. The helmet affixed to an aluminium plate with a 6 degree-of-freedom load cell (45E15; JR3, Woodland, CA, USA). This load cell was connected to a data acquisition device (USB-1608G; Measurement Computing, Norton, MA, USA) and a USB isolator (UHR402; Advantech, Milpitas, CA, USA). Data was sampled at 1000 Hz and raw data was filtered with 4th order Butterworth low pass filter at 50 Hz. A computer monitor was displayed in front of the participants to give real-time visual feedback.

For testing neck flexion force steadiness and force replication sense (accuracy), participants were instructed to push forward flexing their neck and hold steady at 15 Newtons. This target force (15 Newtons) was chosen based on the previous study that reported diminished force steadiness (greater variability) in patients with NP [13]. A custom LabVIEW program (National Instruments, Austin, TX, USA) displayed a trapezoid shape on the monitor which corresponded to a 3-s rest, 3-s ramp up to 15 Newtons, a 10-second at 15 Newtons, a 3-s ramp down to 0 Newtons, and s 3-s rest. During practice trials, participants were asked to follow the shape of this trapezoid using neck flexion force. Participants were asked to hold steady during the 10-second force plateau at 15 Newtons. The average and standard deviation of neck flexion force over an 8-s period was used to represent force steadiness (force output in Newtons; coefficient of variation =standard deviation/average force output). Participants completed the same procedures with and without visual feedback (Figure 3). The force output mean difference between two conditions would be defined as force replication sense. During the no visual feedback trials, an examiner verbally guided participants by stating ‘rest’, ‘ramp-up’, ‘hold steady’, ‘ramp-down’, and ‘rest’. Participants did three trials of each visual feedback condition alternating between conditions with the visual trial occurring first. Force output in each condition as well as coefficient of variation and force replication sense were used for analyses.

**Figure 2.**
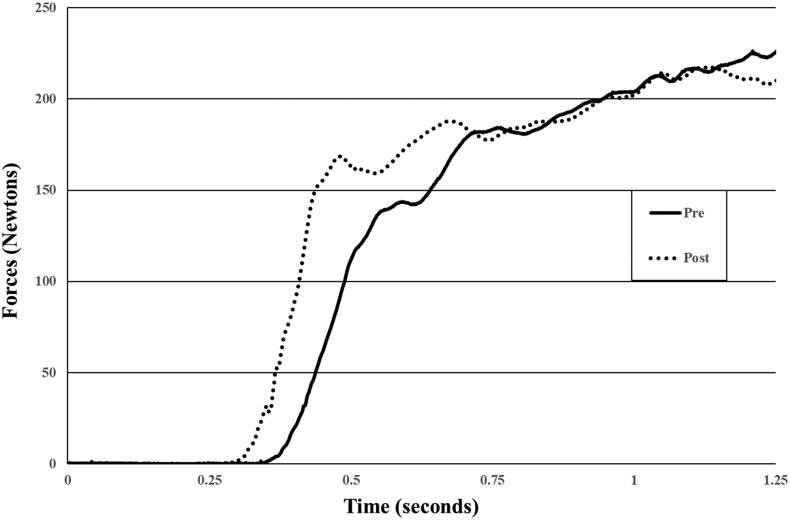
Neck force raw data before (pre) and after (post) neck warm-up. Higher rate of force development was observed while the peak force was mostly unchanged. Visual-motor reaction time was calculated as the time for the force >5 Newtons.

For pre-intervention visual-motor reaction time, peak force, and rate of force development testing, participants sat on the same dynamometer chair and were asked to push against the helmet pad as quick and hard when prompted by a visual arrow on the monitor. A customized LabVIEW program was used to trigger a visual arrow in the forward, backward, right, or left direction at random intervals during each trial. A practice trial at 50% effort was used to allow participants to familiarize themselves with the procedure. For testing, they were instructed to push with as much force as quickly as possible with the directional of the arrow and perform a maximum voluntary isometric contraction for 3 s. The arrow pointed in one of four directions including flexion, extension, and right and left lateral flexion. The computer randomly generated the order of direction of the arrows. The timing of when the first arrow would appear and between arrow prompts varied between 3 and 10 s. Verbal encouragement was provided during the 3-s contraction. Visual-motor reaction time (in milliseconds) was the time from the computer prompt to the participant’s onset of force development at a threshold of 5 Newtons. Peak force was the highest force output in Newtons reported during each trial (Figure 2). The average of three trials was used. Rate of force development (RFD) was calculated as the change in muscle force from the threshold (5 Newtons) to the force output at 50 milliseconds, 100 milliseconds, 150 milliseconds, and 200 milliseconds, respectively (RFD50, RFD10, RFD150, RFD200). Reliability of visual-motor reaction time, peak force, rate of force development variables obtained on the custom device was previously reported and ranged from good to excellent reliability (ICC = 0.406 to 0.948) [16].

**Figure 3.**
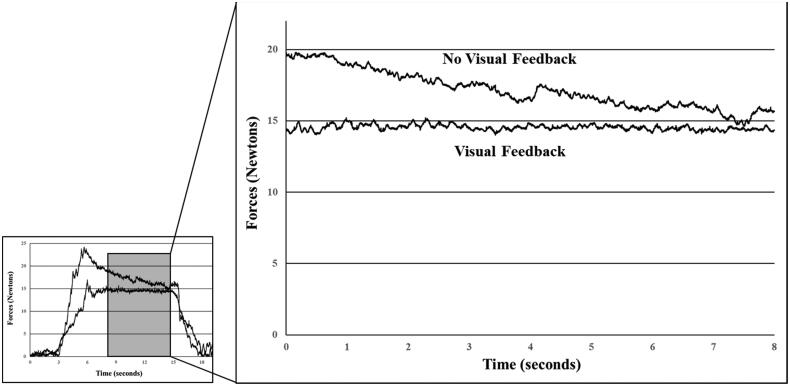
Force steadiness trials with visual feedback trial (force is flat and steady around 15 Newtons) and without visual feedback (force is overshot and gradual declines). force replication sense error was calculated as the average force output difference between two conditions during the Middle 8 s.

### Neck warm-up with isometric contractions

After completion of pre-intervention neck performance testing, participants completed a neck warm-up or bike (sham) warm-up. The 10-minute neck warm-up was performed with the participant seated with an upright posture. The neck warm-up included the following exercises: (A) 5 repetitions of active range-of-motion in flexion and extension, right/left lateral flexions, right/left rotations, and circumduction, (B) three isometric contractions for 5 s in the directions of flexion, extension and right and left lateral flexion at 50%, 75% and 100% effort (2 sets), (C) a cervical rhythmic stabilization/perturbation exercise against examiner provided resistance in random directions (3 times x 15 s), and (D) 5 repetitions of active range-of-motion. On the bike (sham) warm-up day, participants followed the same neck performance testing procedures before and after the sham intervention consisting of 10 min of stationary biking at moderate intensity.

### Procedures study arm #2: Ultrasound-based morphology

For study arm #2, all ultrasound-based measurements were collected using a SL18-5 probe (5-18 MHz bandwidth) and Aixplorer Mach 30 Ultrasound System (SuperSonic Imagine, Inc. USA, Bothell, WA, USA) before a 20-minute rest (sham), pre-, and post- 5-min neck warm-up with isometric contractions. A period of 20 min was allotted between the rest and pre-neck warm-up to serve as control (sham). A 5-min neck warm-up consisted of three isometric contractions each at 50%, 75%, and 100% effort in the flexion direction in supine position and extension in prone (2 sets). An examiner provided instructions, verbal encouragement, and static resistance using a hand-held dynamometer. Participants were asked to hold for 5 s during each isometric contraction. Flexor muscles were scanned immediately after completing the flexion protocol. After a 20-minute rest, the same procedure was repeated for the extensor muscles. The order of testing was randomly assigned.

For acquiring ultrasound images, subjects were either supine or prone position for assessing cervical flexors and extensors, respectively [15]. The cross-sectional area of the longus collis and thickness of the sternocleidomastoid muscles were visualized and saved for later post-processing. Ultrasound measurement for shear wave elastography stiffness of the sternocleidomastoid was obtained at the mid-point of the sternocleidomastoid between the origin and insertion. The probe was oriented longitudinally along the muscle fibres. For the cervical extensor muscles, subjects were prone with a pillow under their chest. Their neck was slightly flexed with their head resting on the table. The cervical extensor muscles included the upper trapezius, splenius capitis, semispinalis capitis, semispinalis cervicis, and multifidi muscles. The probe was oriented transversely to capture the thickness of the extensor muscles at the C5 level slightly lateral from the spinous process. The upper trapezius thickness and shear wave elastography was done in addition to the extensor muscles. The distance between the C7 and the most lateral part of the acromion process was measured first, and then, 2/3 distance from the acromion process was used to scan the upper trapezius. The transducer probe was oriented parallel to the muscle fibres.

**Figure 4.**
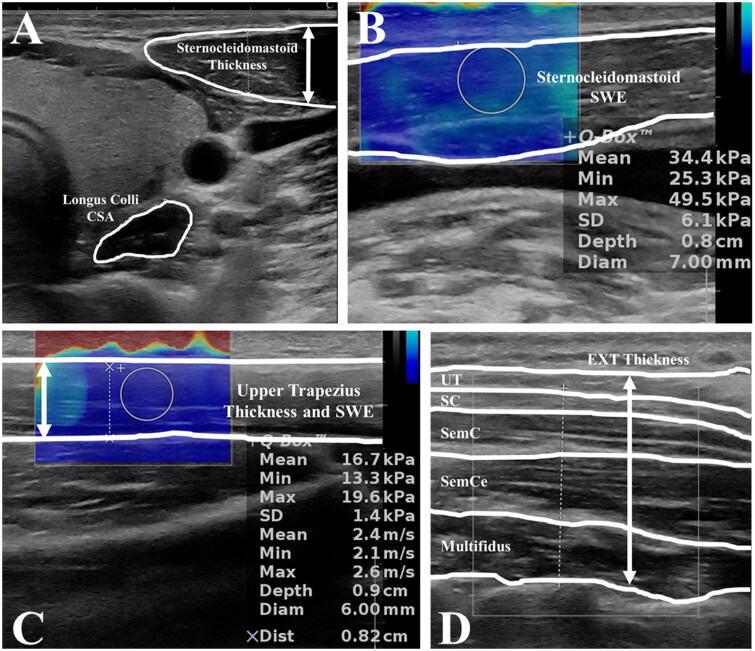
Ultrasound images: A. the sternocleidomastoid thickness and the longus colli cross-sectional area (CSA); B. the sternocleidomastoid shear wave elastography (SWE); C. the upper trapezius thickness and shear wave elastography; and D. the cervical extensors (EXT) thickness.

For post-processing, cross-sectional area of the longus colli was manually traced using the preprogrammed measurement tool within the ultrasound machine (Figure 4). The machine automatically calculated the cross-sectional area in cm^2^. The thickest part, in the anterior-posterior direction in B-Mode, of the sternocleidomastoid was used to measure the thickness in cm. The ultrasound machine recorded several frames of the sternocleidomastoid muscle shear wave elastography stiffness for post-processing. The stiffness was automatically calculated using the software in the machine and expressed in kPa. The thickness of all muscles was measured together as a total cervical extensor muscles’ thickness in cm. Post-processing of the upper trapezius shear wave elastography stiffness was to the same as the sternocleidomastoid. For all measurements, an average of three values was used for statistical analyses. Good to excellent test-retest reliability (ICC = 0.734 − 0.89) was found in all ultrasound-based neck morphology measurements except for the sternocleidomastoid shear wave elastography stiffness (ICC = 0.554) [15].

### Statistical analysis

Descriptive statistics, paired *t*-tests, and Wilcoxon signed-rank tests for non-parametric variables were used to compare neck characteristics before and after each intervention within the same subject for each neck performance variable and ultrasound-based variable. For study arm #1, two-way within-subject analysis of variance (ANOVA) were conducted to identify interactions between interventions (neck warm-up vs. sham) and time (before vs. after). For study arm #2, a one-way within-subject ANOVA was used to determine differences on ultrasound-based morphologies among rest (sham), pre-, and post-intervention. Bonferroni *post hoc* analyses were used if the ANOVA was significant. Significance was set at *p* < 0.05. All statistical analyses were performed using the statistical software SPSS v22 (IBM Corporation, Armonk, NY, USA).

## Results

Descriptive statistics for study arm #1 and arm #2 are shown in Tables 2–3 and 4, respectively. This study was conducted in the early stages of the COVID-19 global pandemic and as a result, 11 subjects who completed the first testing session in the first arm of the study did not return for the second visit due to the COVID-19 related-laboratory closure. As stated in the Introduction and Methods, due to differences in the number of participants in study arm #1 (*n* = 26 for neck warm-up and *n* = 15 for bike/sham), the main effects of neck warm-up and bike warm-up/sham within-subjects were compared using paired t-tests separately. The interaction effects of the neck warm-up and bike warm-up were examined using the 2-way within-subjects ANOVA as originally planned.

**Table 2.** Peak force (PF), visual-motor reaction time (VMRT), and rate of force development (RFD) values in the directions of flexion (flex), extension (ext), and right/left lateral flexions (right and left) before (PRE) and after (POST) neck warm-up and sham warm-up.

	Neck Warm-up		Time	Sham		Time	Interaction
Study Arm #1	PRE	POST	*p*	PRE	POST	*p*	*p*
Flex PF, N	131.6 ± 54.2 (59.1, 290.4)	131.1 ± 51.7 (54.9, 263.8)	0.880	119.1 ± 32.8 (63.0, 173.7)	118.7 ± 29.1 (66.3, 155.8)	0.940	0.976
Flex VMRT, ms	472.3 ± 85.6 (337.0, 601.0)	471.3 ± 102.8 (301.0, 684.0)	0.940	529.9 ± 49.4 (447.0, 626.0)	531.1 ± 69.3 (452.0, 676.0)	0.948	0.921
Flex RFD50, N/s	525.5 ± 427.6 (89.1, 2092.8)	602.1 ± 338.6 (111.5, 1614.1)	0.160	445.9 ± 197.9 (211.8, 840.8)	469.9 ± 189.1 (249.7, 974.8)	0.475	0.483
Flex RFD100, N/s	600.3 ± 391.1 (81.9, 1961.0)	685.7 ± 341.2 (142.8, 1631.9)	**0.022** *	520.4 ± 210.2 (208.6, 873.1)	590.3 ± 209.9 (329.1, 933.5)	0.052	0.769
Flex RFD150, N/s	517.7 ± 309.4 (78.6, 1674.1)	547.2 ± 257.9 (169.5, 1314.2)	0.227	469.6 ± 166.8 (204.7, 790.1)	462.5 ± 41.9 (302.3, 813.3)	0.500	0.913
Flex RFD200, N/s	418.7 ± 211.7 (89.7, 1186.4)	437.8 ± 193.3 (167.4, 1037.0)	0.214	377.4 ± 122.5 (183.4, 552.3)	406.9 ± 127.7 (237.4, 645.8)	0.208	0.692
Ext PF, N	207.8 ± 70.3 (91.3, 375.4)	222.9 ± 76.4 (74.8, 385.0)	0.060	194.7 ± 66.1 (79.2, 346.8)	192.6 ± 69.2 (59.1, 359.5)	0.540	**0.048** *
Ext VMRT, ms	465.9 ± 83.8 (320.7, 629.0)	450.8 ± 76.2 (322.3, 596.0)	0.243	508.3 ± 51.0 (438.0, 579.0)	510.8 ± 63.6 (423.0, 665.0)	0.859	0.376
Ext RFD50, N/s	626.8 ± 344.4 (108.5, 1900.5)	775.2 ± 468.5 (210.0, 2590.8)	**0.003** *	592.9 ± 258.6 (247.1, 1022.8)	569.5 ± 324.4 (227.6, 1539.7)	0.628	**0.019** *
Ext RFD100, N/s	871.4 ± 434.0 (132.7, 2135.2)	1122.7 ± 516.1 (250.3, 2486.3)	**<0.001** *	952.1 ± 469.3 (298.7, 1844.0)	961.5 ± 480.0 (220.2, 1935.7)	0.876	**0.011** *
Ext RFD150, N/s	810.7 ± 404.2 (140.2, 2034.6)	988.5 ± 464.8 (266.6, 2201.8)	**0.001** *	841.4 ± 382.9 (318.5, 1540.2)	818.0 ± 367.8 (226.8, 1639.4)	0.556	**0.007** *
Ext RFD200, N/s	683.6 ± 319.6 (145.8, 1703.7)	820.6 ± 377.0 (245.4, 1806.2)	**0.001** *	716.9 ± 272.9 (294.1, 1229.5)	691.1 ± 267.2 (227.4, 1340.2)	0.418	**0.003** *
Right PF, N	146.2 ± 59.1 (66.2, 330.6)	148.2 ± 51.3 (62.8, 294.1)	0.617	142.8 ± 28.7 (81.6, 180.8)	138.3 ± 36.3 (55.5, 191.5)	0.500	0.399
Right VMRT, ms	426.1 ± 64.2 (318.0, 587.0)	430.7 ± 88.0 (318.3, 703.0)	0.734	489.2 ± 44.7 (410.0, 558.0)	469.5 ± 68.7 (391.0, 675.0)	0.419	0.339
Right RFD50, N/s	549.8 ± 232.1 (185.2, 1216.3)	598.9 ± 281.5 (159.2, 1490.8)	**0.032** *	522.9 ± 229.8 (187.3, 1226.0)	504.9 ± 229.8 (262.8, 1169.7)	0.608	0.091
Right RFD100, N/s	711.0 ± 328.4 (203.8, 1489.7)	789.6 ± 371.8 (195.3, 1706.9)	**0.005** *	709.7 ± 274.4 (231.9, 1173.6)	327.4 ± 84.5 (297.4, 1414.5)	0.745	0.066
Right RFD150, N/s	618.5 ± 268.8 (218.4, 1337.7)	674.7 ± 305.6 (217.2, 1500.6)	**0.004** *	632.5 ± 195.1 (280.0, 950.5)	634.7 ± 220.4 (274.0, 1157.6)	0.945	0.115
Right RFD200, N/s	475.5 ± 184.0 (213.3, 919.5)	513.8 ± 218.4 (236.9, 1082.0)	**0.009** *	480.3 ± 121.1 (321.5, 671.9)	478.0 ± 134.3 (223.9, 714.7)	0.889	0.070
Left PF, N	143.9 ± 53.3 (67.3, 264.1)	152.1 ± 51.2 (47.8, 265.8)	**0.037** *	145.3 ± 32.9 (66.0, 207.0)	137.1 ± 31.7 (65.4, 192.4)	0.066	**0.008** *
Left MVRT, ms	442.2 ± 92.4 (307.0, 714.0)	430.4 ± 94.4 (303.0, 721.0)	0.445	489.6 ± 37.8 (432.0, 537.0)	490.1 ± 47.9 (395.0, 590.0)	0.972	0.586
Left RFD50, N/s	595.2 ± 282.4 (178.0, 1287.8)	626.9 ± 276.7 (215.2, 1232.2)	0.331	553.1 ± 328.0 (258.0, 1553.1)	556.5 ± 266.3 (338.8, 1323.2)	0.927	0.576
Left RFD100, N/s	769.7 ± 406.3 (178.5, 1837.7)	834.6 ± 376.5 (242.0, 1512.9)	0.135	724.2 ± 348.0 (266.3, 1400.3)	769.5 ± 384.8 (365.8, 1534.7)	0.418	0.777
Left RFD150, N/s	653.9 ± 292.6 (194.1, 1395.5)	724.7 ± 300.7 (216.3, 1296.4)	**0.014** *	649.1 ± 217.0 (271.3, 946.8)	656.5 ± 224.7 (348.1, 1090.0)	0.771	0.121
Left RFD200, N/s	475.7 ± 181.8 (167.4, 856.6)	532.7 ± 198.8 (190.7, 913.2)	**0.005** *	480.2 ± 129.7 (236.6, 682.5)	473.0 ± 121.0 (278.7, 673.7)	0.610	**0.008** *

Under the PRE and POST column, means, standard deviations, minimums/maximums (in parentheses) are shown. The column after PRE and POST is representing *p*-values comparing the pre- vs. post-values within each intervention (neck warm-up or sham), based on paired t-tests. The last column represents *p*-values based on the interaction (differences in the pattern of pre-/post-values) between two interventions based on two-way ANOVA analysis.

* represent significant differences (*p* < 0.05).

**Table 3.** Cervical flexion muscular force output and steadiness (coefficient of variation: CoV) with the visual feedback condition (VF) and without visual feedback condition (NoVF), absolute force replication error between force out under VF and NoVF conditions, cervical range-of-motion (ROM) before (PRE) and after (POST) neck warm-up with isometric contractions and sham warm-up.

	Neck Warm-up		Time	Sham		Time	Interaction
Study Arm #1	PRE	POST	*p*	PRE	POST	*p*	*p*
Force Output VF, N	14.9 ± 2.0 (13.6, 24.3)	15.0 ± 1.9 (14.1, 24.4)	0.217	14.6 ± 0.4 (14.1, 15.4)	14.8 ± 0.6 (14.3, 16.7)	0.159	0.672
CoV VF, %	4.7 ± 1.7 (2.3, 10.3)	4.3 ± 1.2 (2.1, 6.4)	0.138	4.2 ± 0.5 (3.0, 4.8)	3.9 ± 0.9 (2.3, 5.1)	0.315	0.663
Force Output NoVF, N	15.2 ± 4.7 (6.5, 28.3)	17.9 ± 7.2 (9.5, 42.5)	**0.011** *	14.4 ± 4.0 (8.0, 21.1)	15.1 ± 3.5 (9.8, 23.3)	0.379	0.171
CoV NoVF, %	6.2 ± 2.0 (3.5, 10.7)	6.9 ± 2.1 (3.4, 12.0)	0.079	8.4 ± 3.1 (2.7, 13.0)	7.8 ± 3.3 (2.5, 14.5)	0.597	0.248
Fore Replication Error, N	3.6 ± 3.2 (0.0, 13.7)	5.1 ± 6.1 (0.0, 27.6)	0.115	2.9 ± 2.1 (0.0, 6.3)	2.3 ± 1.9 (0.3, 6.6)	0.347	0.132
Flex ROM, °	57.1 ± 10.9 (25.0, 70.0)	59.2 ± 9.6 (39.0, 72.6)	0.111	56.1 ± 8.5 (43.0, 73.0)	60.4 ± 9.3 (39.0, 74.0)	**0.036** *	0.329
Ext ROM, °	76.9 ± 10.1 (61.3, 100.0)	78.5 ± 10.0 (59.0, 103.0)	0.194	79.5 ± 11.1 (63.0, 106.0)	79.3 ± 10.8 (62.0, 103.0)	0.862	0.344
Lat. Right ROM, °	43.4 ± 7.5 (31.0, 54.0)	45.4 ± 7.6 (33.0, 60.0)	**0.026** *	44.9 ± 7.7 (28.0, 56.0)	47.9 ± 6.8 (32.0, 60.0)	**0.014** *	0.425
Lat. Left ROM, °	44.8 ± 8.3 (31.6, 61.0)	46.7 ± 8.1 (33.0, 60.0)	**0.003** *	48.1 ± 7.3 (35.0, 63.0)	50.0 ± 6.9 (39.0, 61.0)	0.209	0.964
Rot. Right ROM, °	69.0 ± 7.9 (56.7, 88.0)	70.1 ± 6.4 (60.0, 82.0)	0.277	71.3 ± 8.3 (60.0, 86.0)	72.7 ± 7.6 (55.0, 82.0)	0.309	0.889
Rot. Left ROM, °	70.5 ± 6.7 (60.0, 88.0)	70.8 ± 6.6 (59.0, 90.0)	0.810	73.8 ± 7.0 (64.0, 90.0)	74.7 ± 7.0 (65.0, 90.0)	0.423	0.697

Under the PRE and POST column, means, standard deviations, minimums/maximums (in parentheses) are shown. The column after PRE and POST is representing *p*-values comparing the pre- vs. post-values within each intervention (neck warm-up or sham), based on paired t-tests. The last column represents *p*-values based on the interaction (differences in the pattern of pre-/post-values) between two interventions based on two-way ANOVA analysis.

* represent significant differences.

**Table 4.** Ultrasound-based neck muscle morphological properties: cross-sectional area (CSA), thickness, and/or shear wave elastography (SWE) of the longus collis (LC), sternocleidomastoid (SCM), upper trapezius (up), and the cervical extensors (EXT) at sham (20-minute rest), before (PRE) and after (POST) the maximum voluntary isometric contractions.

				ANOVA
Study Arm #2	Sham	PRE	POST	*p*
LC CSA, cm²	0.75 ± 0.25 (0.39, 1.36)	0.77 ± 0.29 (0.37, 1.52)	0.88 ± 0.36* (0.44, 1.85)	**0.016** *
SCM Thickness, cm	0.75 ± 0.18 (0.45, 1.19)	0.74 ± 0.20 (0.44, 1.11)	0.83 ± 0.19* (0.52, 1.25)	**0.004** *
SCM SWE, kPa	32.2 ± 6.3 (17.6, 43.9)	30.8 ± 8.9 (13.1, 46.9)	33.1 ± 11.6 (18.6, 74.2)	0.560
UT Thickness, cm	1.23 ± 0.28 (0.51, 1.90)	1.12 ± 0.34 (0.60, 1.92)	1.17 ± 0.34 (0.47, 2.01)	0.653
UT SWE, kPa	25.6 ± 11.1 (13.3, 57.0)	25.4 ± 8.3 (11.7, 41.0)	29.4 ± 12.2 (13.9, 64.7)	0.094
EXT Thickness, cm	1.95 ± 0.29 (1.09, 2.40)	2.0 ± 0.34 (1.19, 2.60)	2.1 ± 0.37 (1.44, 3.18)	0.151

Under the PRE and POST column, means, standard deviations, minimums/maximums (in parentheses) are shown. The last column represents *p*-values based on differences among three time points (sham, PRE, and POST) based on one-way ANOVA analysis.

* POST values were significantly larger than sham or PRE values.

For active neck range-of-motion variables, lateral flexion right and left range-of-motion were significantly increased after the neck warm-up (*p* = 0.003–0.026). There were also significant increases in flexion and lateral flexion right range-of-motion after the sham warm-up (*p* = 0.014 − 0.036). Two-way ANOVA revealed that there were no group interactions in range-of-motion variables (*p* = 0.329 −0.964).

For force steadiness variables, subjects generated force above the 15 Newton target in the no visual feedback condition after the neck warm-up (*p* = 0.011). Accordingly, there were trends toward less force steadiness (higher coefficient of variation) and larger force replication sense error after the neck warm-up (*p* = 0.079 − 0.115). There were no significant changes after the sham warm-up (*p* > 0.05).

For visual-motor reaction time, peak force, and rate of force development variables, there were no statistical differences of visual-motor reaction time pre- and post-neck warm-up (*p* = 0.243 − 0.940). For peak force, there was significant increase after the neck warm-up only on left flexion (*p* = 0.037) Left flexion peak force and extension peak force had significant interaction effects (*p* = 0.008 − 0.048), meaning that the neck warm-up was significantly more effective increasing left flexion and extension peak force than the sham warm-up. Meanwhile, there were no significant differences on peak force before and after the sham warm-up (*p* = 0.066 − 0.940). For rate of force development variables, significant increases were observed in several variables after the neck warm-up (*p* < 0.05 on 11 out of 16 variables) while no significant pre- vs. post-intervention effects were observed in the sham warm-up. Interaction (interventions vs. time) effects were significant only in the rate of force development variables (RFD50/100/150/200) in the extension direction and RFD50 in the left lateral flexion direction (*p* = 0.003 − 0.019), meaning that the neck warm-up was more effective increasing extension and left rate of force development when compared to the sham warm-up.

For ultrasound variables, there were significant differences among rest, before, and after the neck isometric contractions on the logus collis cross-sectional area (*p* = 0.016) and the sternocleidomastoid thickness (*p* = 0.004). Specifically, post-hoc analyses revealed that the longus colli cross-sectional area and the sternocleidomastoid thickness were significantly higher at post-neck isometric contractions when compared to either at rest (pre-sham warm-up) or at pre-neck isometric contractions.

## Discussion

### Peak force and rate of force development

We hypothesized that the neck warm-up would yield acute improvements in neck performance characteristics (higher peak force and rate of force development as well as faster visual-motor reaction time). Of these characteristics investigated, the neck warm-up was most effective improving rate of force development as the average improvement was 13% while visual-motor reaction time and peak forces were mostly non-significant (*p* = 0.060 − 0.940) except left lateral flexion peak force (*p* = 0.037). Recent meta-analyses indicated mixed findings on relationship between greater neck strength and mTBI [23,24]. Coaches and athletes should be informed that concussive forces could be mitigated by several factors such as anticipation and stiffness of neck musculatures as well as passive structures such as ligaments [25]. Interestingly, general conditioning programme focusing on the trunk strength including neck strength during pre-season (10 weeks) was effective reducing concussion rates in high school athletes [26]. This is likely that comprehensive warm-up programmes can benefit whole-body motor control and movements that in turn to prepare individuals to increase muscle stiffness and anticipate potential concussive forces. Instead of peak neck forces, individual’s ability to generate peak force (i.e. rate of force development) should be targeted during neck warm-up programmes.

### Post-activation performance enhancement

The observed acute improvements in neck muscular rate of force development were thought to be due to neural adaptions by increased phosphorylation of myosin-light-chains which renders actin-myosin interaction more sensitive to Ca^2+^ released from the sarcoplasmic reticulum, resulting in higher muscular power during evoked twitches or tetanic contractions [27]. The duration and intensity of the neck warm-up (10 min) provided sufficient stimuli without any signs of fatigue or decreases in peak force and rate of force development. Although it is not clear how long the post-activation performance enhancement (PAPE) effects would last, there were mixed findings from as short as a few minutes to as long as 1-48 h after various types of PAPE exercises [8]. Future studies should investigate whether it is possible to keep PAPE effects for longer duration in realistic settings (e.g. 45+ minutes half for a soccer match). Another question was if this phenomenon (PAPE effects) could be used as a part of physical therapy and rehabilitation exercises for faster recovery and return-to-sport for athletes (return-to-duty for military members).

### Effects of pre-participation warm-up on mild traumatic brain injuries and neck pain

There are few prospective studies evaluating effects of warm-up on actual incidences of mTBI and NP. A simple isometric hold in the neck flexion and rotations while rocking/rolling on their back like a rocking chair was effective reducing mTBI incidence, concussive events, and pain during heading among youth soccer players [7]. Similarly, a neck warm-up with neck one maximal isometric contractions in the neck flexion, extension, lateral flexions (right/left) for 10 s in the standing and bear-crawl positions was incorporated into comprehensive injury prevention programme and effective reducing mTBI in youth and adult rugby players [6,28]. In addition to potential protective role against mTBI, neck warm-up programmes might also prevent neck pain. For example, neck resistance training session (20 min per session for 3 times per week for 12 weeks) was implemented in over 700 Australian workers at their workplace and successfully reduced NP cases and severity after 12 weeks [29]. A recent meta-analysis support specific neck exercises were effective reduce the pain intensity among individuals with NP [30].

### Visual-motor reaction time (VMRT)

Contrary to the hypothesis, the neck warm-up with isometric contractions did not improve visual-motor reaction time (VMRT). Slower VMRT is a common clinical sign among concussed athletes and can take several weeks to return to baseline [31]. In our earlier investigations, we identified that female athletes had slower knee and neck visual-motor reaction time as well as lower rate of force development which might be related to higher ACL injury and mTBI in female athletes than their male counterparts [16,32]. As stated in the introduction, early detection to prepare for impact forces (e.g. landing/cutting hard, concussive blunt forces of opponent players, etc.) with early muscle activation could theoretically (based on musculoskeletal modelling) decrease displacement of the head-head-body linkage and reduce linear and angular acceleration at the time of concussive force impact [10,11]. Therefore, different types of exercises might be beneficial to target visual performance. For example, sports vision training such as strobe training (a pair of goggles with flickering lenses) has produced a promising result on visual-motor reaction time [33]. In a small clinical trial, vision training during preseason (∼40 min for 6-7 days/week for 2.5 weeks) and in-season (∼5-10 min for 1 day/week for 16 weeks) was effective reducing incidences of mTBI among college American Football athletes [34]. Additionally, visual-motor reaction time was shown to be a predictive factor separating non-athletes to athletes as well as productive professional athletes (high correlation between vision performance and batting average among professional baseball athletes) [35]. An additional 5-10 min on vision training during neck warm-up would be likely feasible and justified. Stroboscopic training is effective with low volume (2.5-min stroboscopic time: 10-15 min per week for 10 weeks); and the training effect seems to last several weeks after termination of such visual training [33].

### Neck steadiness and force replication accuracy

In our previous investigation, knee force steadiness was examined among athletes who underwent ACL injuries, and the ACL injured leg was showing larger coefficient of variation [36]. In the current investigation, the coefficient of variation did not change before and after the neck warm-up with isometric contractions, rejecting the hypothesis. However, interestingly, the force output (target at 15 Newtons during visual feedback) was significantly increased (over 19%) only during no visual feedback after the neck warm-up. Overshooting of joint position or force replication has been reported in individuals with chronic ankle instability [37] and low back pain [38]. In the current investigation, all participants were healthy with no signs of mTBI/NP. Therefore, the observed force sense deficits after the isometric contractions could likely be explained by muscle thixotropy which describes a history-dependent muscle-contraction [39]. In other words, contractile properties of muscles (actin-myosin cross-bridge and titin) as well as intrinsic muscle fibers (muscle spindles) and potentially Golgi tendon organs remain activated after isometric contractions and increase resting muscle tone/stiffness. This phenomenon can alter and overshoot joint position sense [40] as well as force sensation [41] after repeated bouts of muscle contractions.

While it is not certain whether this overshooting of force is beneficial or detrimental to joint stability and motor control of the cervical spine, coaches and athletes should be aware of this phenomenon. As a countermeasure, isometric contractions-induced proprioception errors could be corrected with stretching in the opposite direction [42]. Although it is beyond scope of the study, athletes or military members who need to perform complex or coordinated motor tasks such as dancing, pitching, shooting, etc. should focus on whole-body warm-up with isometric contractions as well as balance and coordination exercises to improve their proprioception. Several modes of proprioceptive training (e.g. foam rolling, balance training, somatosensory stimulation, etc.) were shown to be effective in clinical patients and healthy individuals [43].

### Ultrasound-based morphology

The current study is one of the first studies to investigate effects of isometric contractions on neck muscular morphology. It was hypothesized that sternocleidomastoid shear wave elastography would increase after isometric contractions due to the thixotropic effects; however, no such significant changes on elastography values were observed, rejected the hypothesis. Higher shear wave elastography stiffness in the sternocleidomastoid and the upper trapezius was observed in individuals with chronic NP [44,45]. Another study highlighted that unaccustomed eccentric contractions of the upper trapezius decreased shear wave elastography stiffness [46]. There are few studies on immediate effects of physical exercises on muscle/tendon shear wave elastography. It would be likely that morphological changes would follow similar history-dependent events (increase shear wave elastography and then gradual decrease back to baseline). More research is needed to confirm the findings with shear wave elastography.

In the current study, the longus colli cross-sectional area and the sternocleidomastoid thickness was significantly increased after isometric contractions while no differences were observed in thickness of the upper trapezius and the extensors. A 10-week programme of craniocervical and cervical flexion resulted in increased cross-sectional area and thickness of the longus colli and increased thickness of the sternocleidomastoid in patients with NP [47]. The current result provided the evidence that simple isometric contractions were effective activating the longus collis and the sternocleidomastoid. This contention was supported by one study that reported improvements in pain, neck disability, endurance, and active range-of-motion after 8 weeks of both craniocervical flexion exercise and neck isometric exercise while craniocervical flexion exercise had advantages on restoring cervical lordosis [48]. Clinically, craniocervical flexion is more technical and difficult to teach to individuals who have never done neck exercises. Future studies can examine how long these changes last and implications for mTBI prevention. Whether different types of exercises or more isometric contractions were needed to activate the cervical extensor muscles should be examined in future investigations.

### Limitations

There were several limitations in the current study. First, the study was designed to examine immediate effects (5–30 min) post-intervention; therefore, it is largely unknown how long these adaptations last. Furthermore, the study focuses on neck performance characteristics and morphology as contributing factors of mTBI and NP, not actual incidence or severity of mTBI and NP. Prospective studies should follow. Second, due to the COVID-19 lockdown, there were 11 participants who only completed the neck warm-up, but, not sham warm-up. More participants in sham warm-up should be included and confirm the result findings on interaction effects between two warm-up programmes (the neck warm-up vs. sham). In study arm #2, a 20-minute rest was used as sham warm-up, and the whole study was completed within one laboratory visit instead of 2 visits during the study arm #1. Therefore, the study design for the study arm #2 was different from the study arm #1. Ideally, we wanted to recruit the same participants for both study arms to further investigate the relationships among neck performance and morphological differences immediately after neck warm-up with isometric contractions. Additionally, the neck ultrasound measurements rely on examiner’s experience. This measurement might not be feasible in a large-scale study with multiple examiners. Simpler methodologies such as neck girth in relation to their body weight or compression-based stiffness measurement device could be used to establish an estimate of neck muscular size and stiffness, respectively.

## Conclusions

Coaches and athletes could include neck warm-up programmes with isometric contractions as a part of whole-body comprehensive warm-up programmes to enhance performance such as sprints, jumps, squats, etc. We identified that the same concept could apply to neck performance as the neck warm-up with isometric contractions was effective in improving some performance characteristics such as RFD and hypertrophy of the surface and deep cervical flexors (the longus colli and the sternocleidomastoid). As more coaches and athletes learn and focus on musculoskeletal injury preventive strategies, they must be aware of various types of pre-participation warm-up exercises. The current findings provide the first evidence that a simple neck warm-up with isometric contractions could be implemented for prior to practices/games for athletes and missions/tactical training for military members. Further refinement would be needed to improve visual-motor reaction time as well as maintain one’s proprioception. Coaches, sports medicine specialists, and researchers collaborate and collectively put efforts to reduce incidence and severity of mTBI and NP.

## Author’s contributions

All authors participated in the conception of the study design, protocol, and manuscript review/final approval. TN, NDS, HW, VCK, STS, RWBC, and DAK participated in data collection and manuscript preparation. TN and NDS participated in data processing and statistical analyses.

## Data Availability

Data are available upon reasonable request.
